# Synthesis, characterization and drug loading property of Monomethoxy-Poly(ethylene glycol)-Poly(ε-caprolactone)-Poly(D,L-lactide) (MPEG-PCLA) copolymers

**DOI:** 10.1038/srep34069

**Published:** 2016-09-28

**Authors:** BingYang Chu, Lan Zhang, Ying Qu, XiaoXin Chen, JinRong Peng, YiXing Huang, ZhiYong Qian

**Affiliations:** 1 Department of Orthopaedic Surgery, Second Affiliated Hospital of Wenzhou Medical University, 109 Xueyuan Road, Wenzhou, 325027, PR China; 2 R&D Center of New Product, Guangdong Zhongsheng Pharmaceutical Co., Ltd., Dongguan, 523325, PR China; 3 State Key Laboratory of Biotherapy and Cancer Center, West China Hospital, Sichuan University and Collaborative Innovation Center for Biotherapy, Chengdu, 610041, PR China

## Abstract

Amphiphilic block copolymers have attracted a great deal of attention in drug delivery systems. In this work, a series of monomethoxy-poly (ethylene glycol)-poly (ε-caprolactone-co-D,L-lactide) (MPEG-PCLA) copolymers with variable composition of poly (ε-caprolactone) (PCL) and poly (D,L-lactide) (PDLLA) were prepared via ring-opening copolymerization of ε-CL and D,L-LA in the presence of MPEG and stannous octoate. The structure and molecular weight were characterized by nuclear magnetic resonance (NMR) and gel permeation chromatography (GPC). The crystallinity, hydrophilicity, thermal stability and hydrolytic degradation behavior were investigated in detail, respectively. The results showed that the prepared amphiphilic MPEG-PCLA copolymers have adjustable properties by altering the composition of PCLA, which make it convenient for clinical applications. Besides, the drug loading properties were also studied. Docetaxel (DTX) could be entrapped in MPEG-PCLA micelles with high loading capacity and encapsulation efficiency. And all lyophilized DTX-loaded MPEG-PCLA micelles except MPEG-PCL micelles were readily re-dissolved in normal saline at 25 °C. In addition, DTX-loaded MPEG-PCLA micelles showed a slightly enhanced antitumor activity compared with free DTX. Furthermore, DTX micelles exhibited a slower and sustained release behavior *in vitro*, and higher DTX concentration and longer retention time *in vivo*. The results suggested that the MPEG-PCLA copolymer with the adjustable ratio of PCL to PDLLA may be a promising drug delivery carrier for DTX.

Biocompatible and biodegradable amphiphilic block copolymers, which consist of hydrophilic and hydrophobic segments, have attracted considerable attention in the biomedical field, especially in drug delivery systems^1^,^2^. These copolymers could easily self-assemble to form nanoscale micelles with a core-shell structure in aqueous solutions. The hydrophobic blocks of the copolymer form a core serving as a container for hydrophobic drugs, while the hydrophilic blocks form a shell to enhance the aqueous stability. In addition, the nanoscale size and the outer shell can reduce the capture of reticuloendothelial system (RES), consequently prolonging their circulation time in blood^3^,^4^,^5^,^6^,^7^. Moreover, by means of passive or/and active targeting effects, these micelles readily accumulate in tumor tissues and then gradually release the loaded drugs locally^8^,^9^.

Poly (ethylene glycol) (PEG) is a most commonly used hydrophilic block with non-toxicity, high safety and eco-friendly feature^10^. While aliphatic polyesters, such as poly (D,L-lactide) (PDLLA), poly(ε-caprolactone) (PCL) and poly(glycolide) (PGA), are a kind of extensively studied hydrophobic polymers with good biodegradability and biocompatibility^11^,^12^. Both of them are commercially available and have been approved for human use by FDA. Thus, various types of PEG-polyester copolymers, such as MPEG-PDLLA^13^,^14^ MPEG-PCL^15^,^16^,^17^ and MPEG-PLGA^18^,^19^ have been developed and applied in drug delivery systems.

MPEG-PCL copolymer has been proved to be a good carrier for hydrophobic drugs delivery^15^,^16^,^17^,^20^. However, owing to the high crystallinity and hydrophobicity of PCL, most of PEG-PCL encounter poor water solubility and slow degradation, which restrict their further clinical application^21^. Previous studies demonstrated that the MPEG_2000_-PCL_2000_ copolymer cannot from micelles in water unless heating up to 60 °C^15^,^16^,^17^. But for clinical application, these kind of drug-loaded copolymer micelles were usually prepared to freeze-dried powder injection for long-term storage and transportation. Before use, normal saline was added to re-dissolve the lyophilized powder. Therefore, the re-dissolved property is very important for clinical application. Considering the high micellar temperature (60 °C) of MPEG-PCL, it is obviously inconvenient in clinic. So the optimization of MPEG-PCL to decrease its crystallinity and increase its water solubility is very significant.

The hydrophobicity and crystallinity can be tailored to achieve desirable water solubility and degradation by tuning the copolymer composition and the ratio among components or modifying the polymer chemically. Previous studies reported that the copolymers of LA/ε-CL, LA/GA, GA/ε-CL at 50:50 monomer compositions present faster degradation and lower crystallinity than corresponding homopolymers^22^,^23^,^24^. Thus, the hydrophilicity and degradation of MPEG-PCL may be adjusted by introducing other monomers. PDLLA has amorphous molecular structure and good water solubility, and a formulation based on MPEG-PDLLA micelles (Genexol^®^-PM) has been approved in clinical trials^25^,^26^,^27^. So, D,L-LA may be a good candidate for ameliorating the property of MPEG-PCL. We assumed that the random copolymerization of ε-CL and D,L-LA in the presence of MPEG as initiator would result in the copolymers (MPEG-PCLA), which may have lower crystallinity, higher hydrophilicity, appropriate degradation rate and drug loading property compared with MPEG-PCL.

In this work, we prepared a series of MPEG-PCLA copolymers with different segment contents of PCL and PDLLA by adjusting the feed ratios of ε-CL to D,L-LA. Then, the crystallinity and hydrophilicity of the prepared MPEG-PCLA copolymers were investigated. The relationships between the thermal property or the hydrolytic degradation behavior and the content of PDLLA segment were also studied. Furthermore, the drug loading capacity, re-dissolved property, cytotoxicity and drug release behavior of lyophilized DTX-loaded MPEG-PCLA micelles were also evaluated.

## Materials and Methods

### Materials

MPEG (M_w_ = 2000), stannous octoate (95%), 3-(4, 5-dimethylthiazol-2-yl)-2, 5-diphenyl-tetrazolium bromide (MTT) were purchased from Sigma-Aldrich (USA). ε-caprolactone (ε-CL) (99%) was purchased from Alfa-Aesar (USA). D,L-lactide (D,L-LA) was bought from Jinan Daigang Biomaterial Co. Ltd (China). Docetaxel (DTX) was purchased from Xieli Pharmaceutical Co., Ltd (China). Docetaxel injection (free DTX) purchased from Hengrui Medicine Co., Ltd (China) was used as positive control. Other chemical reagents were of analytical grade and purchased from Chengdu Kelong Chemicals (China).

3-Dulbecco’s Modified Eagle’s Medium (DMEM), Roswell Park Memorial Institute1640 medium (RPMI 1640) and fetal bovine serum (FBS) were purchased from Invitrogen Corp (USA). Human umbilical vein endothelial Cells (HUVEC), NIH-3T3 Fibroblast cells, human breast cancer cells (MCF-7) and mouse breast cancer cells (4T1) were incubated in medium containing 10% FBS and 1% antibiotics (penicillin- streptomycin, 10000 U/ml) at 37 °C in a humidified incubator containing 5% CO_2_.

Sprague-Dawley (SD) rats were purchased from HFK Bio-Technology. Co., LTD (China) and housed at temperature of 20–22 °C, relative humidity of 50–60% and 12 h light-dark cycles. All rats were quarantined for a week before treatment. All the animal procedures were performed following the protocol approved by the Institutional Animal Care and Treatment Committee of Sichuan University (China). And all rats were treated humanely throughout the experimental period. All methods were carried out in accordance with the approved guidelines (IACUC-S200904-P001).

### Measurements

^1^H-NMR spectra were recorded on a Mercury VX-300 spectrometer (Germany) using tetramethylsilane (TMS) as an internal reference and CDCl_3_ as solvent.

GPC was used to measure the molecular weights of copolymers. GPC analysis was performed on a HLC-8320 GPC system (USA). Tetrahydrofuran (THF) was used as the eluent at a flow rate of 0.6 ml/min, 10 μL of 1% (w/v) sample solutions were injected for each analysis. The molecular weights were calculated based on a universal calibration curve generated with polystyrene standards. The column temperature was set at 40 °C.

Differential scanning calorimeter (DSC) was used to study the thermal properties of the copolymers, performed on a Netzsch DSC 204F1 (Germany).

X-ray diffraction spectra (XRD) were used to measure crystallinity of copolymers, performed on a Philips X’Pert Pro DY1291 (Netherlands) equipped with a Cu-Kα radiation source (λ = 0.1542 nm; 40 kV; 40 mA) at a scanning speed of 2°/min.

Thermos-gravimetric Analysis and the first derivative thermos-gravimetric analysis (TGA/DTG) were carried out by TA Instrument SDT 2960 (USA).

### Synthesis of MPEG-PCLA copolymers

MPEG-PCLA copolymers were prepared by random ring-opening polymerization of ε-CL and D,L-LA, using MPEG as an initiator and stannous octoate as catalyst. In brief, MPEG was heated in vacuum at 100 °C for 2 hour (h) to eliminate the trace amount of water. To avoid the boil of ε-CL, the flask was cooled to room temperature before addition of ε-CL, D,L-LA and stannous octoate (0.3% w/w). The reaction was carried out at 150 °C under nitrogen protection. After 6 h, the reaction mixture was dissolved in dichloromethane and purified by re-precipitation in petroleum. The resulting copolymers were dried in a vacuum oven at 40 °C. The obtained MPEG-PCLA copolymers were dissolved, dialyzed against distilled water and then lyophilized. The purified MPEG-PCLA copolymers were stored in air-tight bottles at −20 °C for further use.

The samples were abbreviated to MPEG-PCLA (the molecular weight of MPEG block–the molecular weight of PCLA block/the content of PCL segment in PCLA block. For MPEG-PCLA (2000–2000/70), the molecular weight of MPEG is 2000 g/mol, the molecular weight of PCLA is 2000 g/mol, and the content of PCL segment in PCLA block is 70%. MPEG-PCLA (2000–2000/100) is equal to MPEG-PCL (2000–2000) (abbreviated as MPEG-PCL), and MPEG-PCLA (2000–2000/0) is equal to MPEG-PDLLA (2000–2000) (abbreviated as MPEG-PDLLA).

### Hydrophilicity

One hundred milligrams of the copolymers were dispersed in 4 ml of distilled water at 25 °C. Later, the samples were stirred (about 50 rpm) at 25 °C for 12 h. Then the hydrophilicity was evaluated by macroscopic observation whether the micelles formed at 25 °C and the solutions were clear and transparent.

### Thermal behavior of the copolymers

Before being submitted for the DSC test and the TGA test, the samples were dried in a vacuum oven at 80 °C for 48 h to eliminate the humidity.

For the DSC test, the samples were first heated to 80 °C and kept for 5 min to eliminate the thermal history. The cooling curves were recorded when the samples were cooled from 80 °C to −20 °C at a rate of 5 °C/min; after the samples was kept at −20 °C for 5 min, the heating curves were recorded from −20 °C to 80 °C at a rate of 5 °C/min. About 5.0–10.0 mg of each sample was tested.

For the TGA/DTG test, all samples (8–10 mg) were heated from 50 °C to 600 °C in an atmosphere of nitrogen at a rate of 10 °C/min. From the TGA and DTG curves, the following characteristics were determined: the temperature at 5% weight loss (*T*_5%_), the temperature at 50% weight loss (*T*_50%_), the temperature at 95% weight loss (*T*_95%_), the temperature at the maximum degradation rate (*T*_max_), weight loss percentage and the temperature at degradation end (*T*_end_) for the corresponding degradation stage, and the residue mass percentage at 500 °C.

### Hydrolytic degradation behavior of the copolymers

The hydrolytic degradation was studied by putting the copolymers (mass = 1.0 g) in 40 ml (a) 1.0 mol/L sodium hydroxide (NaOH) aqueous solution, pH = 13.0; (b) hydrochloric acid (HCl) solution, pH = 1.0; (c) phosphate buffered saline (PBS, 0.1 M, pH = 7.4) at 37 ± 0.5 °C, containing 0.05% sodium azide as an antibacterial agent. At predetermined times, the samples were taken out, dialyzed, frozen and lyophilized to obtain dried degradation products, characterized by ^1^H-NMR, GPC and DSC.

### Preparation and characterization of DTX-loaded copolymer micelles

The thin-film hydration method was used to prepare DTX-loaded copolymer micelles (abbreviated as DTX micelles). Briefly, 10.0 mg DTX and copolymers were co-dissolved in 2 ml of dehydrated alcohol and placed into a round-bottomed flask. Then the solvent was evaporated in rotary evaporator at 60 °C to obtain a thin film. Subsequently, the obtained film was hydrated with 5 ml water under moderate shaking at 60 °C, and DTX-loaded micelles formed via self-assembly. Finally, the solution was filtered with a syringe filter (pore size: 220 nm) (Millex-LG, Millipore Co., USA) to remove non-entrapped drug and lyophilized for further application. Blank micelles were prepared as mentioned above without adding DTX.

The critical micelle concentration (CMC) of MPEG-PCLA (2000–2000/50) was measured using a pyrene fluorescence probe method. Briefly, a series of MPEG-PCLA (2000–2000/50) solutions with a constant pyrene concentration (1.0 × 10^−6^ mol/L) were placed in an ultrasonic bath at 55 °C for 2 h to reach equilibrium. Then the fluorescence spectra were recorded on a luminescence spectrometer (Fluorescence Spectrophotometer LS55, Perkin-Elmer, USA). The emission spectra were scanned from 340 to 450 nm at the excitation wavelength of 333 nm. The CMC value was determined by the curve of fluorescence intensity ratio of I_373_/I_384_ to the micelle concentration.

The drug loading capacity (DLC) and encapsulation efficiency (EE) of DTX micelles were determined as follows. Briefly, the lyophilized DTX micelles were weighed accurately and dissolved in acetonitrile. The amount of DTX in the solution was determined by reverse-phase High Performance Liquid Chromatography (RP-HPLC 1260, Agilent, USA) with a HC-C18 column (4.6 mm × 150 mm, 5 μm) using a mobile phase consisting of acetonitrile/water (55/45, v/v) and UV detection at 232 nm. Finally, DLC and EE of DTX micelles were calculated according to the following formula:


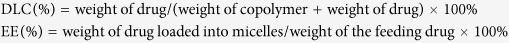


DTX-loaded MPEG-PCLA (2000–2000/50) copolymer micelles were used as an example to evaluate the crystalline properties. Crystallographic assays of DTX powder, the lyophilized DTX-loaded MPEG-PCLA (2000–2000/50) copolymer micelles and the physical mixture of DTX and MPEG-PCLA (2000–2000/50) copolymers was measured by XRD.

The re-dissolved properties of the freeze-dried micelles were investigated. Normal saline was preheated to 25 °C, 40 °C and 60 °C and added into the lyophilized powder, and then stirred (about 50 rpm) to re-dissolve.

The particle size and zeta potential of the prepared DTX micelles were determined by dynamic light scattering (Nano-ZS 90, Malvern, UK). The measurements were performed at 25 °C after equilibration for 2 minutes. All the results were the mean of three test runs, and all data were expressed as the mean ± SD.

The morphology of DTX-loaded MPEG-PCLA (2000–2000/50) micelles was observed under a transmission electron microscopy (TEM H-6009IV, Hitachi, Japan). The samples were diluted with distilled water and placed on a copper grid covered with nitrocellulose. Then the DTX micelles were negatively stained with phosphotungstic acid and dried at room temperature.

### Cytotoxicity of the copolymers and DTX micelles

The cytotoxicity tests of DTX micelles and free DTX were performed on 4T1 cells and MCF-7 cells. The 4T1 or MCF-7 cells were seeded at a density of 1 × 10^4^ cells per well in 100 μL of medium in 96-well plates for 24 h. The cells were then exposed to a series of DTX micelles with different concentrations. After 24 h incubation, the medium was replaced by fresh one, and then 20 μL of MTT solution (5 mg/ml) was added to each well. After incubating for a further 4 h, the MTT solutions were carefully removed, and added 150 μL of Dimethyl sulfoxide (DMSO) to dissolve the MTT formazan crystals. The absorbance was recorded at 570 nm by an ELISA microplate reader (Thermo-Fisher, USA). The relative viability was used to quantify the cytotoxicity, and the control group of copolymer-free culture medium was set as 100% viability. In addition, a cytotoxicity evaluation of the copolymers was conducted on HUVEC cells and NIH-3T3 cells using the MTT method presented above.

### Quantification of cellular uptake

MCF-7 cells at log phase were seeded on 6 well plates with a density of 1 × 10^6^ cells per well. After incubation for 24 h, the media were removed, and cells were exposed to 1 mL serum-free medium containing blank micelles, free DTX, or DTX-loaded MPEG-PCLA (2000–2000/50) micelles at a final concentration of 20 μg/mL, respectively. After incubation for 0 and 4 h, the cells were collected, washed three times with cold phosphate-buffered saline (PBS). Then the cells were added 100 μL of water and freeze-thawed three times to lyse the cells. The cell suspensions were added 300 μL of ethyl acetate, homogenized and then centrifuged at 13000 rpm for 10 min. The DTX amount in the supernatant was measured by HPLC as described above.

### *In vitro* drug release study

*In vitro* drug release was conducted using a dialysis method. Briefly, 1 ml of free DTX or DTX-loaded MPEG-PCLA (2000–2000/50) micelles were placed in dialysis bags (molecular mass cutoff 2000 Da), and then incubated in 40 ml of PBS buffer (pH 7.4) containing 0.5% w/v Tween 80 at 37 °C with gentle shaking (100 rpm). At predetermined time points, the release media were collected and replaced by pre-warmed fresh release media, and then stored at −20 °C for further analysis. After collected all samples, DTX were quantified using HPLC as described above. All the results were the mean of three test runs, and all data were expressed as the mean ± SD.

### *In vivo* pharmacokinetic study

Pharmacokinetics study was performed in healthy SD rats (200 ± 20 g). Twelve rats were fasted overnight, randomly divided into two groups (n = 6) and then administrated intravenously with DTX-loaded MPEG-PCLA (2000–2000/50) micelles or free DTX at a dose of 10 mg DTX/kg body weight, respectively. During the entire experimental period, the rats were supplied with adequate water. At 0.083, 0.25, 0.5, 1, 2, 4, 6, 8 and 12 h after drug administration, blood samples were collected and immediately centrifuged at 6000 rpm for 5 min to obtain the plasma. 100 μL of plasma samples were mixed with 20 μL of paclitaxel solution (10 μg/mL) in methanol as the internal standard and then added 300 μL of ethyl acetate. The mixtures were mixed for 5 min and then centrifuged at 13000 rpm for 10 min. The obtained organic layer was evaporated under nitrogen flow at 40 °C. The residue were reconstituted with 100 μL of the mobile phase consisting of acetonitrile/water (55/45, v/v) and then centrifuged at 13000 rpm for 10 min. 20 μL of the supernatant was injected into HPLC system for HPLC analysis as described above. The pharmacokinetic parameters were calculated using a non-compartmental model by the Drug and Statistics (DAS) software (version 2.1.1, Mathematical Pharmacology Professional Committee, China).

### Statistical analysis

The statistical analysis was carried out with one-way analysis of variance (ANOVA) using the SPSS 15.0 software (Chicago, IL, USA). A *p* < 0.05 was considered as statistically significant difference.

## Results

### Synthesis and characterization of the copolymers

**Figure 1:**
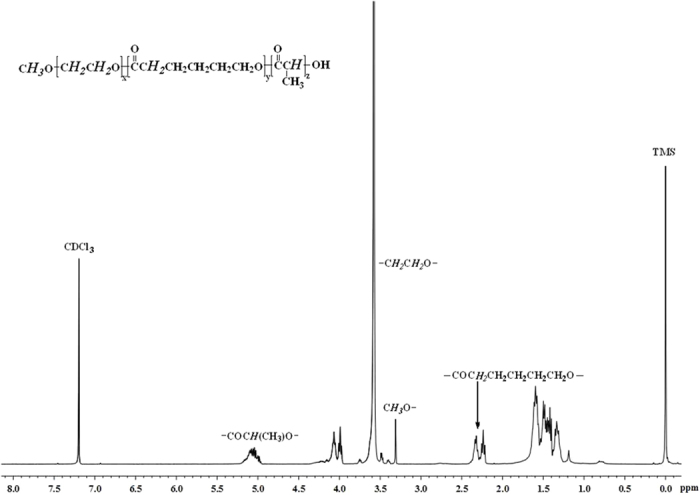
The ^1^H-NMR spectrum of the copolymer in CDCl_3_. (TMS as internal standard substances).

The chemical structure of MPEG-PCLA (2000–2000/50) copolymer was characterized by ^1^H-NMR, as shown in Fig. 1. The peaks around 3.30 ppm belonged to methyl protons of the methoxy units (C***H***_***3***_O-) and the sharp peaks around 3.65 ppm were attributed to methylene protons of the ethylene glycol units (-C***H***_***2***_C***H***_***2***_O-) in MPEG; the peaks around 5.10 ppm and 1.50 ppm belonged to the methylene protons and methyl protons of lactide units (-COCH(CH_3_)O-), respectively; the peaks around 2.30 ppm were attributed to ω-methylene protons of the caprolactone units (-COC***H***_***2***_CH_2_CH_2_CH_2_CH_2_O-), while the two peaks at 1.30 ppm and 1.60 ppm belonged to the repeat ethylene units. All groups of MPEG-PCLA copolymers were identified by corresponding characteristic chemical shifts, indicating successful synthesis of MPEG-PCLA copolymers. The number-average molecular weight (*M*_n_) and block composition of MPEG-PCLA copolymers were determined by using the integral intensities of characteristic peaks at 3.60, 2.30 and 5.10 ppm. The results were listed in Table 1. It can be seen that the measured *M*_n_ and block composition were in good agreement with that designed. GPC was performed to determine the molecular weight distribution (polydispersity index, PDI) (Table 1). All copolymers had a very low value (1.15~1.21), indicating a narrow distribution of PCLA chain length.

**Figure 2:**
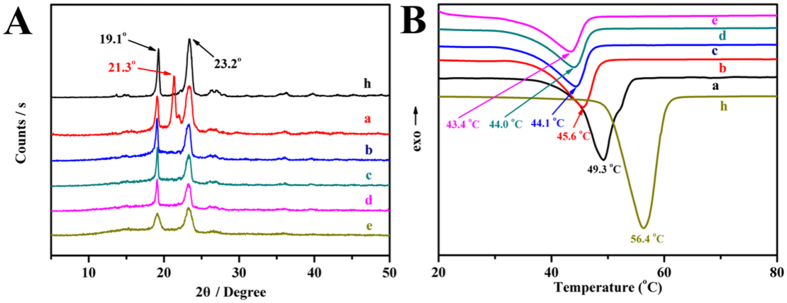
The XRD spectra (**A**) and the DSC curve (**B**) of the copolymers. (a~e, h) represents MPEG-PCLA (2000–2000/100), MPEG-PCLA (2000–2000/70), MPEG-PCLA (2000–2000/50), MPEG-PCLA (2000–2000/30), MPEG-PCLA (2000–2000/0) and MPEG (2000), respectively.

Figure 2A shows the XRD patterns of the copolymers. Two peaks at 19.1° and 23.2° were assigned to the crystalline regions of MPEG block. MPEG-PCLA (2000–2000/100), that is MPEG-PCL, had an obvious crystalline peak at 21.3°, while there was no visible peak of MPEG-PCLA and MPEG-PDLLA (that is MPEG-PCLA (2000–2000/0)) copolymers, indicating the low crystallinity of PCLA and PDLLA segments. DSC analysis further confirmed this result (Fig. 2B). As the content of PDLLA segments increased, melting peaks of the copolymers decreased accordingly, indicating that their crystallinity decreased too.

**Figure 3:**
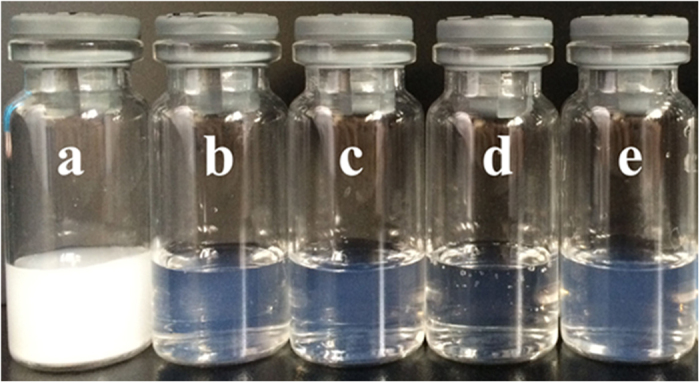
The water solubility of the copolymers at 25 °C. (**a~e**) Represents MPEG-PCLA (2000–2000/100), MPEG-PCLA (2000–2000/70), MPEG-PCLA (2000–2000/50), MPEG-PCLA (2000–2000/30) and MPEG-PCLA (2000–2000/0), respectively.

Figure 3 shows that the appearances of the copolymers in water at 25 °C. Obviously, the solution of MPEG-PCL was turbid. While other copolymers formed micelles with good clarity and faint opalescence. These results indicated that the prepared MPEG-PCLA copolymers had great hydrophilicity compared with MPEG-PCL.

### Thermal degradation in an atmosphere of nitrogen

**Figure 4:**
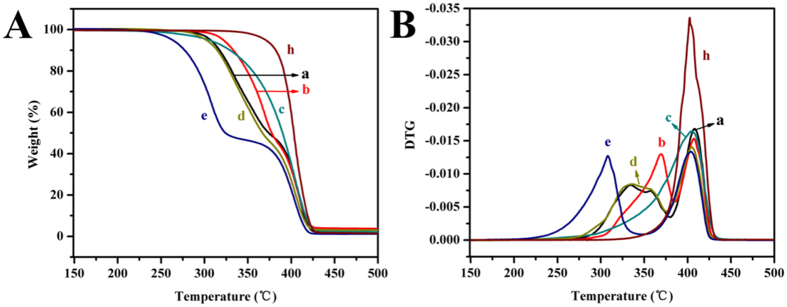
The TGA curves (**A**) and the corresponding DTG curves (**B**) of the copolymers in an atmosphere of nitrogen. (a~e, h) represents MPEG-PCLA (2000–2000/100), MPEG-PCLA (2000–2000/70), MPEG-PCLA (2000–2000/50), MPEG-PCLA (2000–2000/30), MPEG-PCLA (2000–2000/0) and MPEG (2000), respectively.

The thermal stability of the copolymers was studied by means of TGA. The TGA and DTG curves of copolymers were showed in Fig. 4, and the characteristic values from TGA curves were summarized in Table 2.

All copolymers showed a similar two-stage degradation pattern in nitrogen except MPEG-PCLA (2000–2000/50), which showed a one-stage degradation pattern, as shown in Fig. 4B. The degradation of MPEG segment mainly occurred at *ca.* 405 °C, while the degradation of polyester segments occurred at relative low temperature. With the content of PDLLA segment in PCLA blocks increasing, *T*_*d,5%*_and *T*_*d,1 max*_increased firstly and then decreased, while reached maximum at the equal ratio (50:50) of PDLLA to PCL. The results indicated that MPEG-PCL was more stable to heat than MPEG-PDLLA, but both were less stable than MPEG-PCLA. Therefore, the thermal stability was concerned with the content of PDLLA in the PCLA block; and MPEG-PCLA copolymer with an intermediate ratio (50:50) of PCL to PDLLA was the most stable.

### Hydrolytic degradation behavior of the copolymers

**Figure 5:**
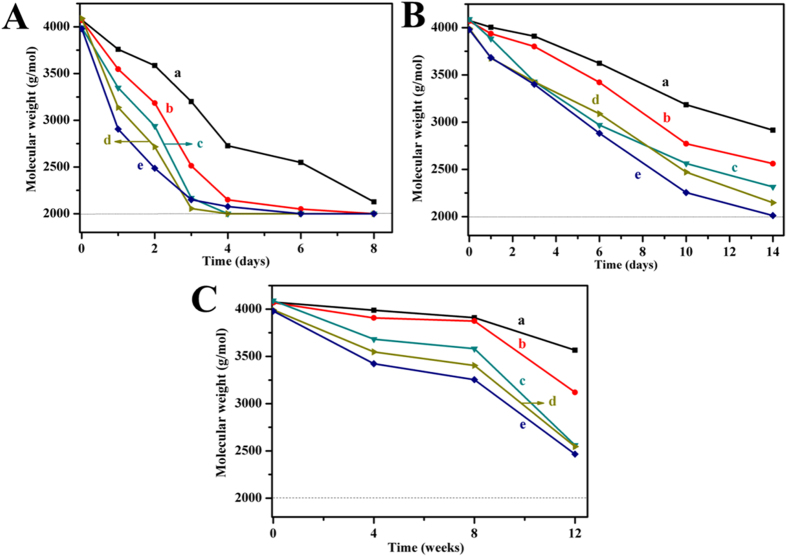
The *M*_n_ changes of the copolymers before and after degradation in basic solutions (pH = 13.0) (**A**), acidic solutions (pH = 1.0) (**B**) and PBS (pH = 7.4) (**C**). (a~e) represents MPEG-PCLA (2000–2000/100), MPEG-PCLA (2000–2000/70), MPEG-PCLA (2000–2000/50), MPEG-PCLA (2000–2000/30) and MPEG-PCLA (2000–2000/0), respectively.

The hydrolytic degradation behaviors of all copolymers in basic (pH = 13.0), acidic (pH = 1.0) and PBS (pH = 7.4) solutions were examined at 37 °C. Figure 5 shows the changes of molecular weight measured by ^1^H-NMR during degradation. Although the molecular weight of all copolymers decreased due to the degradation, the degradation rates were different. In the same degradation medium, the residual molecular weight of MPEG-PCL was the biggest and that of MPEG-PDLLA was the smallest after the same degradation time, while the MPEG-PCLA copolymers were in between both. In addition, the residual molecular weights of the MPEG-PCLA copolymers decreased as the content of PDLLA segments increased.

**Figure 6:**
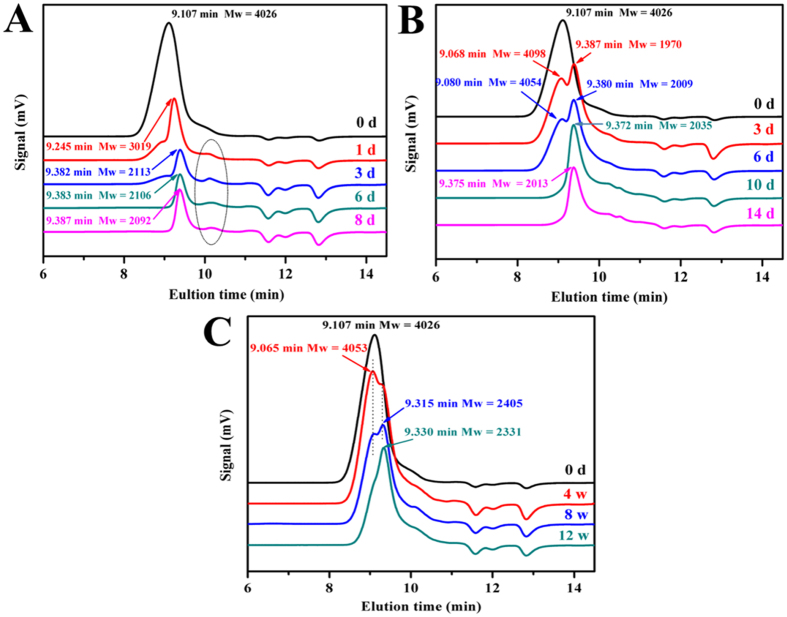
The GPC curves of MPEG-PCLA (2000–2000/50) copolymer before and after degradation in basic solutions (pH = 13.0) (**A**), acidic solutions (pH = 1.0) (**B**) and PBS (pH = 7.4) (**C**).

The degradation of the copolymers was also affected by the degradation medium. The molecular weights of MPEG-PCLA (2000–2000/50) were nearly 2000 or 2350 g/mol after 8 days in basic solution (pH = 13.0) or 14 days in acidic solution (pH = 1.0), but 2550 g/mol after 12 weeks in PBS (pH = 7.4). It’s because that the hydrolysis of the ester bonds in MPEG-PCLA copolymer could be accelerated in the presence of acid or base, while PBS kept the solution to be neutral and therefore slowed down the degradation. Figure 6 exhibits the GPC profiles of MPEG-PCLA (2000–2000/50) at different degradation solutions. The increased elution time indicated that the molecular weight decreased. The signal of MPEG segment gradually appeared and increased. Meanwhile the signal of PCLA segments disappeared after 8 days in basic solution or 14 days in acidic solution, but still existed after 12 weeks in PBS. These results indicated that the degradation measured by GPC were in accordance with that of ^1^H-NMR.

**Figure 7:**
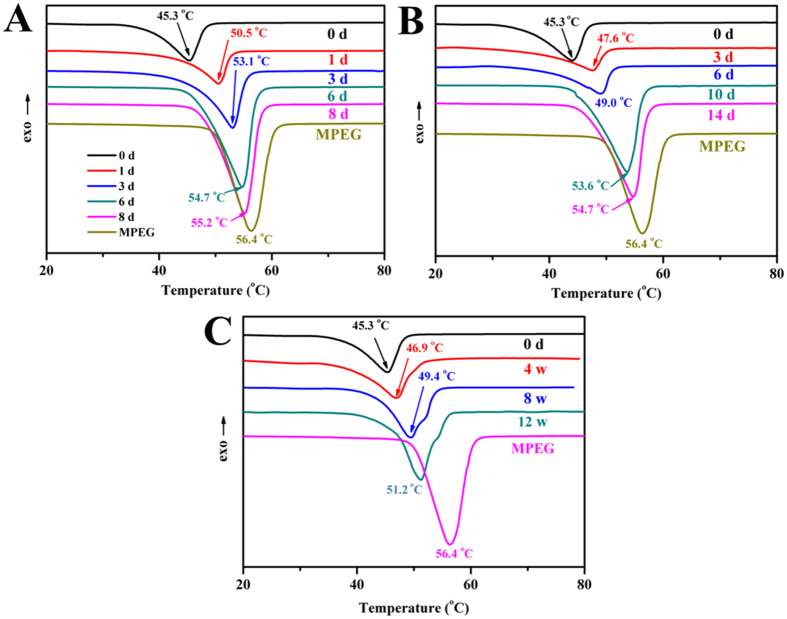
The DSC curves of MPEG-PCLA (2000–2000/50) copolymer before and after degradation in basic solutions (pH = 13.0) (**A**), acidic solutions (pH = 1.0) (**B**) and PBS (pH = 7.4) (**C**).

Figure 7 shows the DSC curves of the copolymers before and after degradation. The melting points of MPEG-PCLA (2000–2000/50) and MPEG were 45.3 °C and 56.4 °C, respectively. As the degradation proceeded, the melting points increased gradually and were gradually closer to that of MPEG, indicating that MPEG gradually appeared and increased.

### Preparation and characterization of DTX-loaded copolymeric micelles

The DTX micelles were prepared by a thin-film hydration method. During the evaporation process to remove dehydrated alcohol, DTX and copolymer distributed as homogenous amorphous substance. After water or normal saline added, all copolymers could self-assemble to form core-shell structural micelles with DTX encapsulated in core at appropriate temperature.

**Figure 8:**
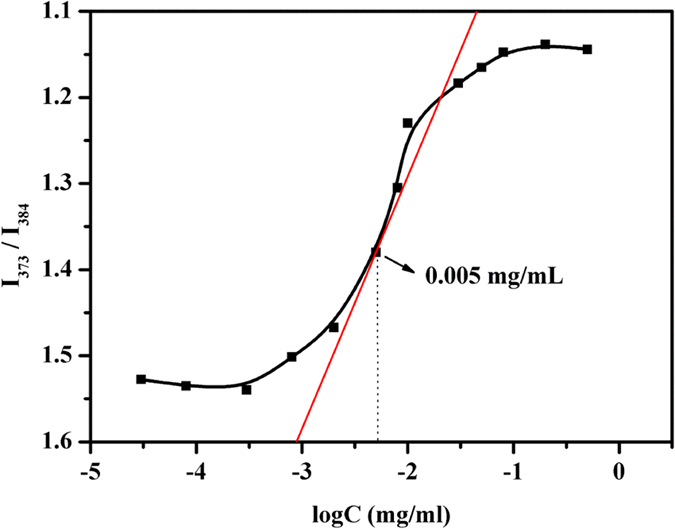
The CMC curve of MPEG-PCLA (2000–2000/50) copolymer micelles.

The CMC curve of MPEG-PCLA (2000–2000/50) micelles was shown in Fig. 8. The CMC value was determined by the point which had the largest inclined rate. According to this, the CMC value of MPEG-PCLA (2000–2000/50) micelles was about 0.005 mg/mL.

To investigate the drug loading capacity of MPEG-PCLA copolymers, various drug/copolymer weight ratios were designed in the preparation of micelles, and the results were shown in Table 3. For each copolymer, the increased drug loading capacity and the decreased encapsulation efficiency were presented with the drug/copolymer feed ratio rising. The encapsulation efficiency was kept at a high level ( > 90%) at the designed feeding DTX/copolymers weight ratio. In addition, the drug loading capacity and encapsulation efficiency of MPEG-PCLA copolymers had no difference with that of MPEG-PCL and MPEG-PDLLA, indicating the excellent drug loading property.

**Figure 9:**
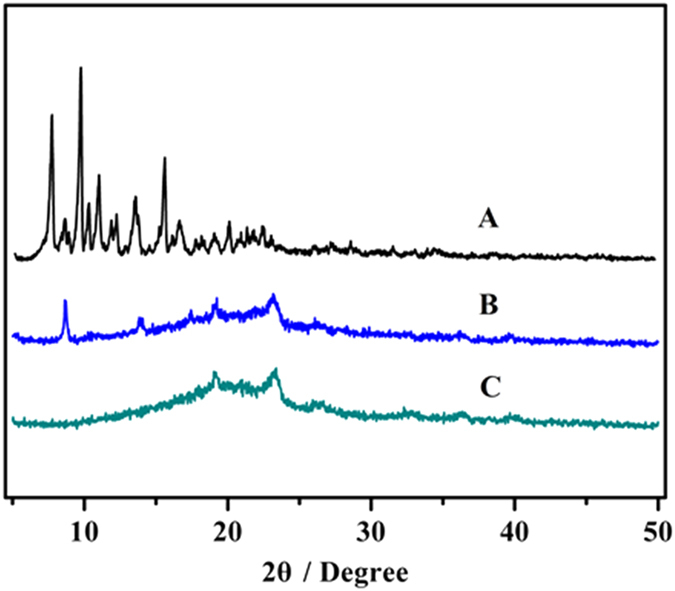
X-ray diffraction spectra of DTX powder (**A**), physical mixture of DTX and MPEG-PCLA (2000–2000/50) copolymers (**B**) and lyophilized DTX-loaded MPEG-PCLA (2000–2000/50) copolymer micelles (**C**).

Crystallographic analysis was performed by XRD as presented in Fig. 9. Compared with the XRD spectrum of DTX and the physical mixture, the lack of characteristic diffraction peak in the spectra of lyophilized DTX-loaded MPEG-PCLA (2000–2000/50) micelles indicated that DTX was completely entrapped in the micelles.

**Figure 10:**
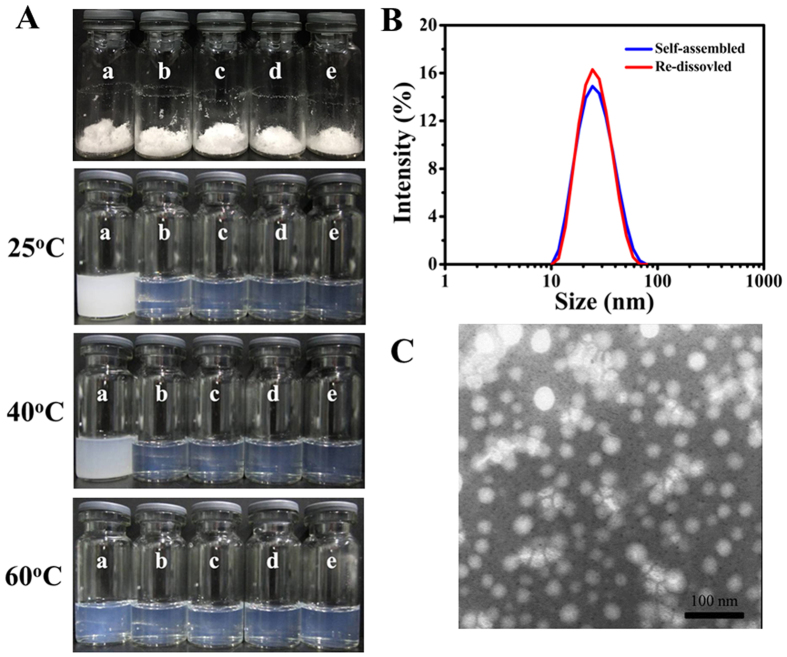
(**A**) Photographs of freeze-dried and re-dissolved DTX micelles based on different copolymers in normal saline at 25 °C, 40 °C and 60 °C. (a~e) represents DTX micelles of MPEG-PCLA (2000–2000/100), MPEG-PCLA (2000–2000/70), MPEG-PCLA (2000–2000/50), MPEG-PCLA (2000–2000/30) and MPEG-PCLA (2000–2000/0), respectively; (**B**) Particle size distribution of freshly prepared and re-dissolved freeze-dried DTX-loaded MPEG-PCLA (2000–2000/50) copolymer micelles; (**C**) TEM images of re-dissolved freeze-dried DTX-loaded MPEG-PCLA (2000–2000/50) copolymer micelles.

Considering long-term storage and easy of transportation, the DTX micelles were usually used as lyophilized powder. Normal saline was added to re-dissolve the lyophilized micelles when used. As shown in Fig. 10A, all lyophilized micelles were powder forms without any excipient and could be completely re-dissolved as clear fluid without any surfactants or additives in normal saline at 60 °C. But the lyophilized DTX-loaded MPEG-PCL micelles were turbid slurry at 40 °C and 25 °C, while the other lyophilized DTX micelles were still transparent. Moreover, the particle size did not almost any change between lyophilized and re-dissolved DTX-loaded MPEG-PCLA (2000–2000/50) micelles at 25 °C (Fig. 10B). The particle size and zeta potential of the re-dissolved micelles were shown in Table 4. All micelles had smaller particle size than 25 nm and were almost not charged. TEM images showed that the re-dissolved micelles were still monodisperse and spherical particles with uniform size (Fig. 10C). The results further suggested that MPEG-PCLA copolymers could successfully solve the poor hydrophilicity of MPEG-PCL.

### Cytotoxicity assay

**Figure 11:**
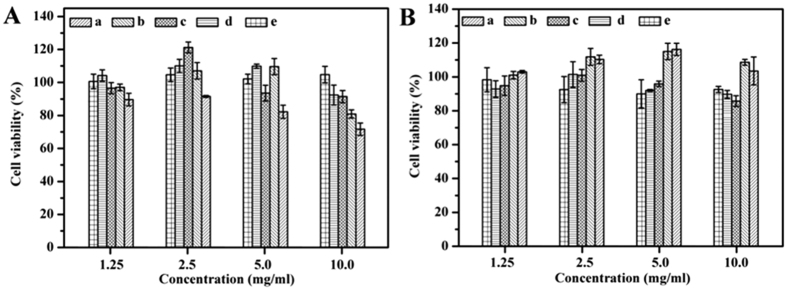
Cytotoxicity of the copolymers on HUVEC cells (**A**) and NIH-3T3 cells (**B**) detected by MTT assay, (means ± SD, n = 6). (a~e) represents MPEG-PCLA (2000–2000/100), MPEG-PCLA (2000–2000/70), MPEG-PCLA (2000–2000/50), MPEG-PCLA (2000–2000/30) and MPEG-PCLA (2000–2000/0), respectively.

The cytotoxicity of the copolymers was evaluated by a cell viability assay using HUVEC and NIH-3T3 cells for 24 h, respectively. According to Fig. 11A,B, there were more than 80% of HUVEC and NIH-3T3 cells viable in almost all the copolymer solutions at a concentration of 10.0 mg/ml. The cytotoxicity studies suggested that MPEG-PCLA copolymers had good biocompatibility as MPEG-PCL and MPEG-PDLLA. Therefore, MPEG-PCLA copolymers could be regarded as safe drug delivery carriers.

**Figure 12:**
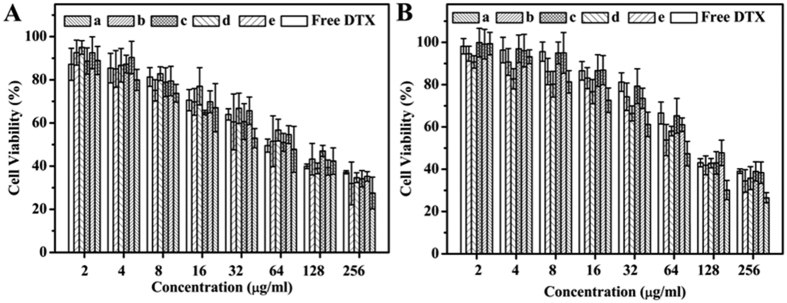
Cytotoxicity of DTX-loaded copolymer micelles on 4T1 cells (**A**) and MCF-7 cells (**B**) detected by MTT assay, (means ± SD, n = 6).(a~e) represents MPEG-PCLA (2000–2000/100), MPEG-PCLA (2000–2000/70), MPEG-PCLA (2000–2000/50), MPEG-PCLA (2000–2000/30) and MPEG-PCLA (2000–2000/0), respectively.

To evaluate the cytotoxicity of DTX-loaded MPEG-PCLA micelles, 4T1 cells and MCF-7 cells were exposed to a series of DTX micelles with different concentrations. Figure 12A,B showed that free DTX and all DTX micelles significantly inhibited the growth of 4T1 cells and MCF-7 cells in a dose-dependent manner. All DTX micelles had similar IC_50_ and were slightly lower than free DTX (Table 5). The results demonstrated the encapsulation of DTX into MPEG-PCLA micelles kept the cytotoxicity compared with DTX-loaded MPEG-PCL micelles, and slightly enhanced the cytotoxicity than free DTX.

### Quantification of cellular uptake

**Figure 13:**
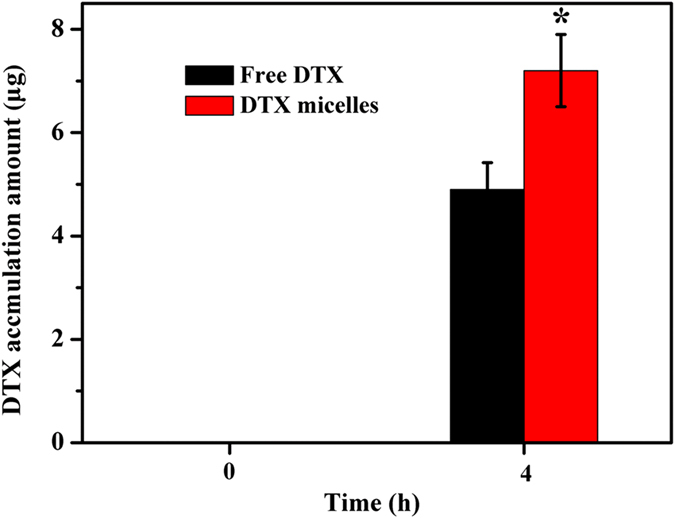
The amount of DTX accumulated in MCF-7 cells. DTX micelles in figure refer to DTX-loaded MPEG-PCLA (2000–2000/50) micelles. *p < 0.05, compared with free DTX group.

The cellular uptake of DTX by MCF-7 cells was quantified using HPLC method. As shown in Fig. 13, DTX uptake by MCF-7 cells was 4.93 ± 0.52 μg in free DTX group, while significantly more DTX (7.28 ± 0.69 μg, *p* < 0.05) accumulated in MCF-7 cells in DTX-loaded MPEG-PCLA (2000–2000/50) micelles group. The results indicated that DTX micelles could improve the uptake of DTX by MCF-7 cells.

### *In vitro* drug release behavior

**Figure 14:**
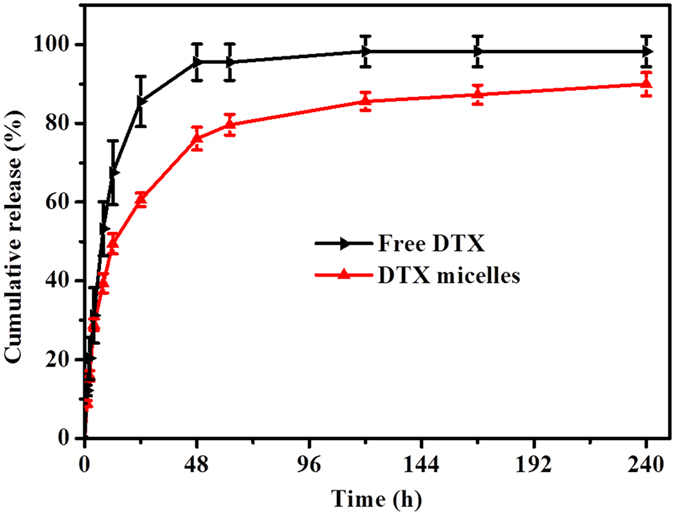
*In vitro* release profiles of DTX from free DTX and DTX-loaded MPEG-PCLA (2000–2000/50) micelles in PBS solution containing 0.5% (w/v) Tween 80 at pH 7.4, the error bars represent the standard deviation (n = 3). DTX micelles in figure refer to DTX-loaded MPEG-PCLA (2000–2000/50) micelles.

The *in vitro* drug release behavior of DTX from free DTX and DTX-loaded MPEG-PCLA (2000–2000/50) micelles was shown in Fig. 14. In the first 24 hours, compared with 85.6% of DTX released from free DTX, only 60.6% of DTX were released from DTX-loaded MPEG-PCLA (2000–2000/50) micelles. Besides, the release of DTX from the micelles also exhibited slow and sustained release behavior even up 240 hours. These controlled release behavior may be in favor of reducing the leakage of DTX in the systemic circulation and avoiding the damage to the healthy tissue.

### *In vivo* pharmacokinetic study

**Figure 15:**
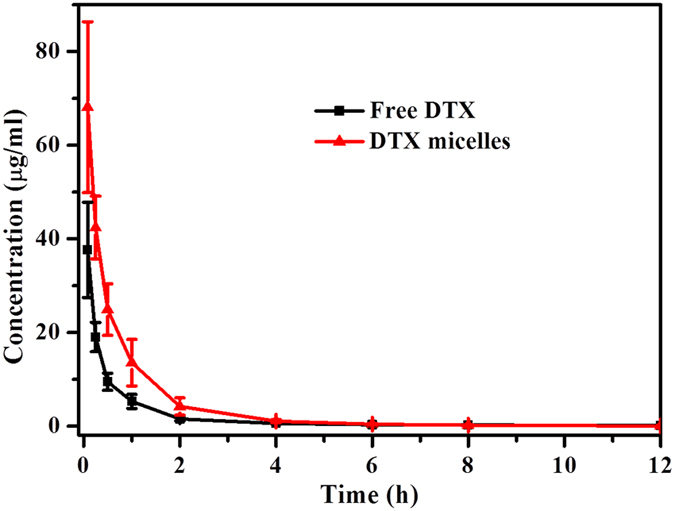
Plasma concentration-time profiles of DTX after intravenous injection of free DTX and DTX-loaded MPEG-PCLA (2000–2000/50) micelles in rat. Error bars represent the standard deviation (n = 3). DTX micelles in figure refer to DTX-loaded MPEG-PCLA (2000–2000/50) micelles.

After intravenously administration of free DTX and DTX-loaded MPEG-PCLA (2000–2000/50) micelles, the concentrations of DTX in plasma were measured, the plasma concentration-time profiles were shown in Fig. 15 and the main parameters were presented in Table 6. The concentrations of DTX were significantly higher in DTX-loaded MPEG-PCLA (2000–2000/50) micelles group compared with free DTX group, and the peak concentrations (C_max_) were 68.10 ± 18.26 mg/L versus 37.63 ± 10.17 mg/L (*p* < 0.05). For bioavailability, the area under the plasma concentration-time curve (AUC) of DTX in DTX-loaded MPEG-PCLA (2000–2000/50) micelles group was 2.2 times higher than that in free DTX group (46.96 ± 10.49 mg/L^*^h versus 21.60 ± 4.56 mg/L^*^h, *p* < 0.05). Furthermore, the corresponding total body clearance (CL) of DTX decreased from 0.48 ± 0.11 L/h/kg (free DTX) to 0.22 ± 0.05 L/h/kg (DTX-loaded MPEG-PCLA (2000–2000/50) micelles) (*p* < 0.05). Compared with free DTX, the higher C_max_ and AUC and decreased CL of DTX-loaded MPEG-PCLA (2000–2000/50) micelles suggested that the clearance of DTX in plasma could be prevented by polymeric micelles and then improved the bioavailability.

## Discussion

Polyether-polyester block copolymers are amphiphilic, biodegradable and easy to prepare, and have been extensively applied in drug delivery systems. In our previous work, MPEG-PCL had been used to deliver paclitaxel and tacrolimus. However, owing to high crystallinity and hydrophobicity of the PCL segment, MPEG_2000_-PCL_2000_ cannot dissolve in water at room temperature unless heating up to 60 °C, which restrict its further application in clinic^15^,^16^,^17^,^21^,^28^.

In this work, we tailored the properties of MPEG-PCL copolymer by the random ring-opening copolymerization of ε-CL and D,L-LA. The prepared MPEG-PCLA copolymers have controllable molecular weight and segment content, and easily synthesized as designed by adjusting the feed ratio of ε-CL and D,L-LA. Introducing PDLLA into PCL segments decreased the crystallinity and improved the water solubility. It’s known that PCL has high crystallinity, but PDLLA is amorphous^29^. When PLA was copolymerized with PCL segments, the ordered crystalline structure of PCL segment was disturbed, thus leading to an amorphous molecular structure of their copolymer (PCLA)^30^. For MPEG-PCLA, the water solubility is mainly affected by the hydrophobic segments because hydrophilic MPEG has good solubility in water. The crystalline segments prevent water diffusion into the polymer, but the amorphous segments can facilitate water diffusion. Thus the introduction of PDLLA into MPEG-PCL copolymers will result in the decrease of the crystallinity, and hence the water molecules were more easily to diffuse through their copolymers (MPEG-PCLA), leading their copolymers to easily dissolve in water at room temperature.

The thermal stability of MPEG-PCLA increased at first and then decreased with the increase of the PDLLA content, reaching a highest value at an equal content (50:50) of PCL and PDLLA segment. The result is also in accordance with the studies that PCL is more stable to heat than PLA^22^,^30^,^31^,^32^,^33^,^34^. The thermal degradation is mainly affected by the chain structure of the copolymers. The change of the chain structure may result in the difference of the degradation behavior. Therefore, we can synthesize MPEG-PCLA copolymers with appropriate thermo-stability via adjusting the ratio of PDLLA to PCL. Moreover, the weight losses of PCLA segments at stage 1 were ca.52~55% (Table 2), showing that the contents of PCLA segments were 52~55% in the MPEG-PCLA copolymers. These results were in accordance with the ^1^H-NMR data.

The hydrolytic degradation rate of MPEG-PCLA copolymers also increased as the content of PDLLA in PCLA block increased. In addition, the degradation rate also depends on the degradation medium, basic and acid medium remarkably accelerated the degradation of MPEG-PCLA compared with PBS. The hydrolytic degradation of MPEG-PCLA copolymers is mainly due to the breakage of the ester bonds of the polyester blocks, which depends on the content of PDLLA segment and the degradation medium^28^,^35^,^36^. The high content of PDLLA segment leads to the accelerated degradation. One reason for this result is the fact that the copolymers with higher PDLLA content possessed poorer crystallinity, which made water easier access to the ester bonds of MPEG-PCLA copolymers. The other reason is that the PDLLA chains had higher ester group content than PCL chain, which made the copolymers of higher PDLLA content more hydrophilic than those of lower PDLLA content. So, lower crystallinity and higher content of ester bonds of those copolymers with higher PDLLA content led to a rapider hydrolytic degradation. The degradation medium also affects the hydrolytic degradation. Free OH^−^ and H^+^ are the catalyst of the breakage of the eater bonds and can accelerate the degradation of the copolymers. At the same time, the acidic production resulting from the degradation of the PDLLA segment will also self-catalyzed the copolymers. Therefore, we can easily prepare MPEG-PCLA copolymers with appropriate hydrolytic degradation property for desired clinical application via adjusting the ratio of PDLLA to PCL.

As a drug delivery carrier, the drug loading properties is the most important parameters. MPEG-PCL and MPEG-PDLLA copolymers had been demonstrated as excellent carrier for DTX^13^,^37^. The CMC of MPEG-PCLA (2000–2000/50) micelles was about 0.005 mg/mL, which is in the same order of magnitude as that of MPEG-PDLLA (0.008 mg/mL) and MPEG-PCL (0.01 mg/mL)^38^. The low CMC is in favor of keeping the micelles stability and reducing the leakage in systemic circulation^39^. We also investigated the DTX loading property of MPEG-PCLA copolymer. The results showed there was no significant difference on the drug loading capacity and encapsulation efficiency of all prepared DTX micelles. However, the re-dissolved phenomenon of the lyophilized micelles was different. The lyophilized DTX-loaded MPEG-PCLA micelles were readily dissolved in normal saline at 25 °C, whereas the lyophilized DTX-loaded MPEG-PCL micelles required higher temperature (*ca.*60 °C). This good re-dissolved property of DTX-loaded MPEG-PCLA micelles makes it convenient for clinical application. Besides, all DTX formulation exhibited a significant suppression on the growth of 4T1 and MCF-7 cells. But in comparison with free DTX, the DTX micelles had slightly lower IC_50_ and enhanced cytotoxicity. Through cellular uptake study, we found the encapsulation of DTX into micelles could enhance the uptake by cells compared with free DTX, which may explain the enhanced cytotoxicity of DTX micelles. Furthermore, compared with free DTX, DTX micelles exhibited a slower and sustained release behavior *in vitro* and the improved DTX concentration and retention time *in vivo*. These drug release behaviors may be in favor of improving the therapeutic effect through the longer circulation time, the lower DTX leakage and the more accumulation of DTX in tumors via EPR effect.

## Conclusion

In this paper, we successfully prepared MPEG-PCLA copolymers with different segment ratios of PCL to PDLLA by adjusting the feed ratio of ε-CL to D,L-LA. The introduction of PDLLA into PCL block lowers the crystallinity and improves hydrophilicity compared with MPEG-PCL. While the thermal stability increased at first and then decreased, and the MPEG-PCLA copolymer with an intermediate ratio (50:50) of PCL to PDLLA was the most stable to heat. Higher content of PDLLA segment led to rapider hydrolytic degradation, while basic and acidic solutions also accelerated the hydrolytic degradation of MPEG-PCLA copolymers. So, according to the need, MPEG-PCLA copolymers with specific thermal and hydrolytic degradation properties can be prepared via adjusting the feed ratio of PCL to PDLLA. Besides, all copolymers exhibited good biocompatibility, high drug loading capacity and encapsulation efficiency. In addition, compared with free DTX, DTX-loaded MPEG-PCLA micelles showed increased cellular uptake, slightly enhanced cytotoxicity, controlled release behavior *in vitro* and improved pharmacokinetics *in vivo*. More importantly, MPEG-PCLA copolymers solved the poor water solubility of MPEG-PCL, which is beneficial to the clinical applications. Therefore, MPEG-PCLA copolymers with adjustable ratio of PCL to PDLLA may be a promising drug delivery system and deserve further study.

## Additional Information

**How to cite this article**: Chu, B.Y. *et al.* Synthesis, characterization and drug loading property of Monomethoxy-Poly(ethylene glycol)-Poly(ε-caprolactone)-Poly(D,L-lactide) (MPEG-PCLA) copolymers. *Sci. Rep.* **6**, 34069; doi: 10.1038/srep34069 (2016).

## Figures and Tables

**Table 1:** ^1^H-NMR characterizations and GPC measurements of the copolymers.

Code	Sample	Designed			^1^H-NMR			GPC
		Total *M*_n_	%CL^v^	%LA^v^	*M*_n_	%CL^w^	%LA^w^	PDI^x^
**a**	MPEG-PCLA (2000–2000/100)^y^	4000	100	0	4074	100	0	1.179
**b**	MPEG-PCLA (2000–2000/70)	4000	70	30	4069	69.5	30.5	1.178
**c**	MPEG-PCLA (2000–2000/50)	4000	50	50	4090	50.7	49.3	1.204
**d**	MPEG-PCLA (2000–2000/30)	4000	30	70	3993	33.9	66.1	1.159
**e**	MPEG-PCLA (2000–2000/0)	4000	0	100	3980	0	100	1.194

^v^The designed hydrophobic segment composition.

^w^The hydrophobic segment compositions determined by ^1^H-NMR.

^x^Polydispersity index (PDI, *M*_w_/*M*_n_) determined by GPC.

^y^MPEG-PCLA (2000–2000/100) is equal to MPEG-PCL (2000–2000), while MPEG-PCLA (2000–2000/0) is equal to MPEG-PDLLA (2000–2000).

**Table 2:** The TGA data of the copolymers at a heating rate of 10 °C/min in an atmosphere of nitrogen.

Sample	*T*_*d,5%*_ (°C)	Stage1			Stage 2		*T*_*d,95%*_ (°C)	Residue at 500 °C (%)
		*T*_d.1max_ (°C)	*T*_d_._1end_ (°C)	Weight loss (%)	*T*_d_._2max_ (°C)	Weight loss (%)		
MPEG-PCLA(2000–2000/100)	307.0	334.3	380.3	52.3	408.3	45.8	419.7	1.9
MPEG-PCLA(2000–2000/70)	321.7	369.7	385.0	55.1	407.3	41.2	423.0	3.7
MPEG-PCLA(2000–2000/50)	308.3	—*	—*	—*	404.7	98.0	422.7	2.0
MPEG-PCLA(2000–2000/30)	302.0	337.0	376.5	54.7	405.0	42.3	419.0	3.0
MPEG-PCLA(2000–2000/0)	263.0	308.0	347.5	53.4	404.0	45.4	415.0	1.2
MPEG	370.5	—*	—*	—*	402.5	98.6	422.7	1.4

^*^“—” No data was obtained.

**Table 3:** Drug loading capacity (DLC) and encapsulation efficiency (EE) of the DTX micelles based on various copolymers.

	Sample	Drug/copolymer mass ratio in feed (w/w)			
		2/98	5/95	8/92	12/88
DLC(%)	MPEG-PCLA(2000–2000/100)	1.92 ± 0.07	4.79 ± 0.19	7.66 ± 0.34	10.67 ± 0.26
	MPEG-PCLA(2000/2000/70)	1.97 ± 0.04	4.83 ± 0.22	7.33 ± 0.04	9.65 ± 0.14
	MPEG-PCLA(2000/2000/50)	1.98 ± 0.04	4.90 ± 0.06	7.80 ± 0.02	10.71 ± 0.68
	MPEG-PCLA(2000/2000/30)	1.95 ± 0.05	4.90 ± 0.02	7.63 ± 0.45	9.15 ± 0.34
	MPEG-PCLA(2000–2000/0)	1.94 ± 0.02	4.90 ± 0.23	7.68 ± 0.20	11.15 ± 0.08
EE(%)	MPEG-PCLA(2000–2000/100)	96.19 ± 3.47	95.75 ± 3.72	95.77 ± 4.19	88.95 ± 2.13
	MPEG-PCLA(2000/2000/70)	98.29 ± 1.95	96.69 ± 4.40	91.61 ± 0.51	80.39 ± 1.19
	MPEG-PCLA(2000/2000/50)	99.07 ± 1.90	97.92 ± 1.21	97.54 ± 0.26	89.29 ± 5.70
	MPEG-PCLA(2000/2000/30)	97.37 ± 2.45	97.98 ± 0.43	95.38 ± 5.61	76.23 ± 2.83
	MPEG-PCLA(2000–2000/0)	97.21 ± 0.85	97.91 ± 4.51	95.97 ± 2.47	92.88 ± 0.64

**Table 4:** The particle size and zeta potential of the DTX micelles based on various copolymers.

Sample	Size (nm)	Zeta potential (mV)
DTX/MPEG-PCLA(2000–2000/100)	24.207 ± 0.068	−0.493 ± 0.038
DTX/MPEG-PCLA(2000/2000/70)	23.627 ± 0.072	−0.278 ± 0.022
DTX/MPEG-PCLA(2000/2000/50)	24.060 ± 0.061	−0.394 ± 0.030
DTX/MPEG-PCLA(2000/2000/30)	23.750 ± 0.029	−0.622 ± 0.048
DTX/MPEG-PCLA(2000–2000/0)	21.913 ± 0.202	−0.438 ± 0.057

**Table 5:** The IC_50_ values of the DTX micelles on 4T1 cells and MCF-7 cells tested by MTT assay.

Sample	IC_50_ value (μg/ml)	
	4T1	MCF-7
Free DTX	93.5	132.7
DTX/MPEG-PCLA(2000–2000/100)	53.1	73.2
DTX/MPEG-PCLA(2000/2000/70)	79.7	122.2
DTX/MPEG-PCLA(2000/2000/50)	72.9	124.8
DTX/MPEG-PCLA(2000/2000/30)	86.3	121.5
DTX/MPEG-PCLA(2000–2000/0)	70.2	109.1

**Table 6:** Pharmacokinetic parameters after intravenous administration of DTX formulations in rats *p < 0.05, compared with free DTX group.

Parameters	Free DTX	DTX- mPEG-PCLA (2000–2000/50) micelles
AUC(mg/L*h)	21.60 ± 4.56	46.96 ± 10.49*
C_max_(mg/L)	37.63 ± 10.17	68.10 ± 18.26*
MRT(h)	1.61 ± 0.18	1.13 ± 0.21*
t1/2(h)	1.11 ± 0.13	0.78 ± 0.15*
CL(L/h/kg)	0.48 ± 0.11	0.22 ± 0.05*
V(L/kg)	0.78 ± 0.21	0.25 ± 0.08*

## References

[b1] Tian H., Tang Z., Zhuang X., Chen X. & Jing X. Biodegradable synthetic polymers: preparation, functionalization and biomedical application. Prog. Polym. Sci. 37, 237–280 (2012).

[b2] Larson N. & Ghandehari H. Polymeric conjugates for drug delivery. Chem. Mater. 24, 840–853 (2012). 22707853 10.1021/cm2031569 PMC3374380

[b3] Gaucher G. *et al.* Block copolymer micelles: preparation, characterization and application in drug delivery. J. Control. Release 109, 169–188 (2005). 16289422 10.1016/j.jconrel.2005.09.034

[b4] Kwon G. S. & Kataoka K. Block copolymer micelles as long-circulating drug vehicles. Adv. Drug Deliver. Rev. 16, 295–309 (1995).

[b5] Rijcken C., Schiffelers R., van Nostrum C. & Hennink W. Long circulating biodegradable polymeric micelles: towards targeted drug delivery. J. Control. Release 132, e33–e35 (2008).

[b6] Kwon G. S. & Kataoka K. Block copolymer micelles as long-circulating drug vehicles. Adv. Drug Deliver. Rev. 64, 237–245 (2012).

[b7] Chu B. *et al.* PEG-derivatized octacosanol as micellar carrier for paclitaxel delivery. Int. J. Pharm. 500, 345–359 (2016). 26794876 10.1016/j.ijpharm.2016.01.030

[b8] Petros R. A. & DeSimone J. M. Strategies in the design of nanoparticles for therapeutic applications. Nat. Rev. Drug Discov. 9, 615–627 (2010). 20616808 10.1038/nrd2591

[b9] Peer D. *et al.* Nanocarriers as an emerging platform for cancer therapy. Nat. Nanotechnol. 2, 751–760 (2007). 18654426 10.1038/nnano.2007.387

[b10] Torchilin V. P. Structure and design of polymeric surfactant-based drug delivery systems. J. Control. Release 73, 137–172 (2001). 11516494 10.1016/s0168-3659(01)00299-1

[b11] Cameron D. J. & Shaver M. P. Aliphatic polyester polymer stars: synthesis, properties and applications in biomedicine and nanotechnology. Chem. Soc. Rev. 40, 1761–1776 (2011). 21082079 10.1039/c0cs00091d

[b12] Hillmyer M. A. & Tolman W. B. Aliphatic Polyester Block Polymers: Renewable, Degradable, and Sustainable. Accounts Chem. Res. 47, 2390–2396 (2014). 10.1021/ar500121d 24852135

[b13] Lee S.-W. *et al.* Development of docetaxel-loaded intravenous formulation, Nanoxel-PM™ using polymer-based delivery system. J. Control. Release 155, 262–271 (2011). 21704664 10.1016/j.jconrel.2011.06.012

[b14] Blanco E. *et al.* β-Lapachone-containing PEG–PLA polymer micelles as novel nanotherapeutics against NQO1-overexpressing tumor cells. J. Control. Release 122, 365–374 (2007). 17574288 10.1016/j.jconrel.2007.04.014 PMC2064869

[b15] Gong C. *et al.* Improving antiangiogenesis and anti-tumor activity of curcumin by biodegradable polymeric micelles. Biomaterials 34, 1413–1432 (2013). 23164423 10.1016/j.biomaterials.2012.10.068

[b16] Wang Y. *et al.* Micelles of Methoxy Poly (ethylene glycol)–Poly (ε-caprolactone) as a Novel Drug Delivery Vehicle for Tacrolimus. J. Biomed. Nanotechnol. 9, 147–157 (2013). 23627041 10.1166/jbn.2013.1489

[b17] Wang C. *et al.* Characterization, pharmacokinetics and disposition of novel nanoscale preparations of paclitaxel. Int. J. Pharm. 414, 251–259 (2011). 21596124 10.1016/j.ijpharm.2011.05.014

[b18] Hu X., Han R., Quan L.-H., Liu C.-Y. & Liao Y.-H. Stabilization and sustained release of zeylenone, a soft cytotoxic drug, within polymeric micelles for local antitumor drug delivery. Int. J. Pharm. 450, 331–337 (2013). 23587966 10.1016/j.ijpharm.2013.04.007

[b19] Guo P. *et al.* *In vitro* and *in vivo* evaluation of APRPG-modified angiogenic vessel targeting micelles for anticancer therapy. Int. J. Pharm. 486, 356–366 (2015). 25840274 10.1016/j.ijpharm.2015.03.067

[b20] Wang Y. *et al.* Pharmacokinetics and disposition of nanomedicine using biodegradable PEG/PCL polymers as drug carriers. Curr. Drug Metab. 13, 338–353 (2012). 22443531 10.2174/138920012800166490

[b21] Li S., Chen X., Gross R. & McCarthy S. Hydrolytic degradation of PCL/PEO copolymers in alkaline media. J. Mater. Sci.-Mater. Med. 11, 227–233 (2000). 15348037 10.1023/a:1008920326988

[b22] Fernández J., Etxeberria A., Ugartemendia J. M., Petisco S. & Sarasua J.-R. Effects of chain microstructures on mechanical behavior and aging of a poly (L-lactide-co-ε-caprolactone) biomedical thermoplastic-elastomer. J. Mech. Behav. Biomed. Mater. 12, 29–38 (2012). 22659093 10.1016/j.jmbbm.2012.03.008

[b23] Vert M., Li S. & Garreau H. More about the degradation of LA/GA-derived matrices in aqueous media. J. Control. Release 16, 15–26 (1991).

[b24] Kasperczyk J., Li S., Jaworska J., Dobrzyński P. & Vert M. Degradation of copolymers obtained by ring-opening polymerization of glycolide and ɛ-caprolactone: A high resolution NMR and ESI-MS study. Polym. Degrad. Stabil. 93, 990–999 (2008).

[b25] Lim W. *et al.* Phase I pharmacokinetic study of a weekly liposomal paclitaxel formulation (Genexol^®^-PM) in patients with solid tumors. Ann. Oncol. mdp315 (2009). 10.1093/annonc/mdp315 19633055

[b26] Kim T.-Y. *et al.* Phase I and pharmacokinetic study of Genexol-PM, a cremophor-free, polymeric micelle-formulated paclitaxel, in patients with advanced malignancies. Clin. Cancer Res. 10, 3708–3716 (2004). 15173077 10.1158/1078-0432.CCR-03-0655

[b27] Kim D.-W. *et al.* Multicenter phase II trial of Genexol-PM, a novel Cremophor-free, polymeric micelle formulation of paclitaxel, with cisplatin in patients with advanced non-small-cell lung cancer. Ann. Oncol. 18, 2009–2014 (2007). 17785767 10.1093/annonc/mdm374

[b28] Li S. *et al.* Hydrolytic degradation of poly (oxyethylene)-poly-(ε-caprolactone) multiblock copolymers. J. Appl. Polym. Sci. 68, 989–998 (1998).

[b29] Kim K. *et al.* Control of degradation rate and hydrophilicity in electrospun non-woven poly (D, L-lactide) nanofiber scaffolds for biomedical applications. Biomaterials 24, 4977–4985 (2003). 14559011 10.1016/s0142-9612(03)00407-1

[b30] Nalampang K., Molloy R. & Punyodom W. Synthesis and characterization of poly (L-lactide-co-ε-caprolactone) copolymers: influence of sequential monomer addition on chain microstructure. Polym. Advan. Technol. 18, 240–248 (2007).

[b31] Jiang T., He F. & Zhuo R.-X. Synthesis, characterization and enzymatic degradation of novel biodegradable copolymers of 5-allyloxy-1, 3-dioxan-2-one with ε-caprolactone. Polym. Degrad. Stabil. 98, 325–330 (2013).

[b32] Fernández J., Etxeberria A. & Sarasua J.-R. Synthesis, structure and properties of poly(L-lactide-co–caprolactone) statistical copolymers. J. Mech. Behav. Biomed. Mater. 9, 100–112 (2012). 22498288 10.1016/j.jmbbm.2012.01.003

[b33] Fernández J. *et al.* Synthesis and characterization of poly (l-lactide/ε-caprolactone) statistical copolymers with well resolved chain microstructures. Polymer 54, 2621–2631 (2013).

[b34] Fernández J., Etxeberría A. & Sarasua J. R. Effects of repeat unit sequence distribution and residual catalyst on thermal degradation of poly(l-lactide/ε-caprolactone) statistical copolymers. Polym. Degrad. Stabil. 98, 1293–1299 (2013).

[b35] Qian H., Bei J. & Wang S. Synthesis, characterization and degradation of ABA block copolymer of L-lactide and ε-caprolactone. Polym. Degrad. Stabil. 68, 423–429 (2000).

[b36] Qian Z., Li S., He Y., Zhang H. & Liu X. Hydrolytic degradation study of biodegradable polyesteramide copolymers based on ε-caprolactone and 11-aminoundecanoic acid. Biomaterials 25, 1975–1981 (2004). 14741611 10.1016/s0142-9612(03)00604-5

[b37] Ostacolo L. *et al.* *In vitro* anticancer activity of docetaxel-loaded micelles based on poly (ethylene oxide)-poly (epsilon-caprolactone) block copolymers: Do nanocarrier properties have a role? J. Control. Release 148, 255–263 (2010). 20816710 10.1016/j.jconrel.2010.08.006

[b38] Chen L. *et al.* Which polymer is more suitable for etoposide: A comparison between two kinds of drug loaded polymeric micelles *in vitro* and *in vivo*? Int. J. Pharm. 495, 265–275 (2015). 26325322 10.1016/j.ijpharm.2015.08.043

[b39] Chu B. *et al.* PEG-derivatized octacosanol as micellar carrier for paclitaxel delivery. Int. J. Pharm. 500, 345–359 (2016). 26794876 10.1016/j.ijpharm.2016.01.030

